# Exploring the associations of generalized trust, climate change conspiracy beliefs and freecycling: Empirical evidence from 34 cultures

**DOI:** 10.1111/bjop.70058

**Published:** 2026-01-23

**Authors:** Algae K. Y. Au, Jacky C. K. Ng, Sylvia Xiaohua Chen

**Affiliations:** ^1^ The Hong Kong Polytechnic University Hong Kong China

**Keywords:** climate change conspiracy beliefs, freecycling, generalized trust, pro‐environmental behaviours

## Abstract

This study examined the relationships between generalized trust, climate change conspiracy beliefs and freecycling – a community‐based free‐item sharing pro‐environmental behaviour. It also explored the role of societal factors in relation to participation in freecycling, as well as how they are associated with these relationships. Using a panel method, we conducted an online survey with 16,773 participants, stratified by age, gender and region across 34 countries/societies. Key findings indicate that generalized trust and, unexpectedly, climate change conspiracy beliefs are positively associated with freecycling participation. Our exploratory results show that freecycling is more prevalent in developing societies, characterized by stronger beliefs in reward for application and religiosity, a lesser emphasis on uncertainty avoidance and a preference for short‐term over long‐term orientation. Cross‐level moderation analysis indicates that generalized trust is more strongly linked to freecycling in developing societies; its association with freecycle giving is also stronger in cultures with lower reward for application. Climate change conspiracy beliefs are more strongly linked to freecycling in societies with lower uncertainty avoidance. By addressing gaps in the existing literature, particularly the need for cross‐cultural comparisons, our research offers valuable insights into the construct of freecycling. As we navigate the complexities of hyperconsumerism and climate change conspiracy beliefs, scepticism towards mainstream narratives may sometimes be associated with individuals seeking alternative, grassroots solutions. Promoting freecycling could encourage sustainability, strengthening community connections and empowering individuals to take direct action in response to their doubts, potentially contributing to a more resilient and environmentally aware society.

## BACKGROUND

The modern world is dominated by hyperconsumerism (Lipovetsky, 2010), an unsustainable behaviour characterized by the excessive acquisition of material goods to satisfy non‐basic needs (Dimitrova et al., 2022). Over the past two decades, however, online marketplaces such as Craigslist and Gumtree have grown rapidly, enabling peer‐to‐peer trading of used goods. This sharing economy not only signifies a shift towards sustainable consumption but also acts as an antidote to hyperconsumerism (Liu et al., 2020).

Alongside this, freecycling – a non‐monetary, non‐reciprocal community sharing economy – has emerged (Liu et al., 2020). Freecycling involves giving away second‐hand items for free with no expectation of return and no obligations for receiving. While the primary focus of freecycling is to minimize waste, it also benefits others and builds community (Aptekar, 2016). With 5000 local groups and 11 million global freecyclers, The Freecycle Network (TFN) – the key player in this global movement – keeps over 1000 tons of usable items out of landfills daily (The Freecycle Network [TFN], 2024). Considering the existence of numerous other freecycling groups across the globe, the collective number of freecyclers and the overall impact of freecycling on both the community and the environment could be even more substantial.

Despite the unique contribution of freecycling in waste reduction, research on this emerging and community‐based pro‐environment behaviour remains scarce. Most of the existing freecycling research is small‐scale and focused on a single culture (Aptekar, 2016; Nelson et al., 2007), except for some big data analyses using data mining techniques (Norbutas & Corten, 2018). The majority of these studies have been conducted in Western cultures (Aptekar, 2016; Nelson et al., 2007; Norbutas & Corten, 2018), with limited exceptions in Turkey (Özcelik & Kaplan, 2021) and Hong Kong (Lou, 2019). Moreover, most participants in these studies were freecycle givers rather than receivers, leaving the experiences of receiving largely underexplored (Aptekar, 2016). This highlights a critical gap in freecycling research, particularly the need for large‐scale, cross‐cultural studies that encompass both Western and non‐Western samples and examine both giving and receiving.

Moreover, while the positive associations of generalized trust (Gupta & Ogden, 2009; Gür, 2020; Mannemar Sønderskov, 2011) and negative associations of climate change conspiracy beliefs (Biddlestone et al., 2022; Jolley & Douglas, 2014) with everyday life pro‐environmental behaviours are well‐documented, their associations with freecycling remain underexplored. To address this gap, our preregistered study investigated the associations of generalized trust (Aptekar, 2016; Norbutas & Corten, 2018) and climate change conspiracy beliefs (Chan, Tam, & Hong, 2023; Wood, 2017) with freecycle giving and receiving among 16,773 participants across 34 countries/societies. Beyond these preregistered analyses, we conducted additional exploratory analyses examining the role of societal factors in freecycling participation and their connections to the relationships of generalized trust and climate change conspiracy beliefs with freecycling. By studying freecycling with multicultural data, we aim to understand how individual characteristics and cultural contexts relate to freecycle participation.

### Freecycling: A community‐driven approach to sharing and reusing

Initiated by TFN in 2003, freecycling is a community‐driven global pro‐environmental movement that aims to reduce waste and alleviate landfill burdens while benefiting individuals and fostering community building (TFN, 2024). Freecycling involves joining local online groups on websites or social media platforms, where members can give away and request items for free, typically within their own neighbourhood for pick‐up convenience (Aptekar, 2016). What sets freecycling apart from other commercialized sharing economies is its non‐monetary and non‐reciprocal nature, making it a sharing economy in its purest form. Unlike platforms that involve monetary exchanges or direct reciprocity, freecycling operates on the principle of gifting to strangers without expecting direct compensation, emphasizing environmentalism and waste reduction (Aptekar, 2016; Lou, 2019; Norbutas & Corten, 2018).

What differentiates freecycling from everyday life pro‐environmental behaviours is its community‐oriented nature. Freecycling creates a sense of community, bringing together environmentally like‐minded neighbours who share a commitment to protecting the environment (Aptekar, 2016). Private‐sphere pro‐environmental behaviours such as recycling or reducing energy consumption at home, in contrast, do not inherently involve community building (Tsai et al., 2021). Moreover, freecycling enables participants to identify themselves as both givers and receivers, aligning with the sharing economy's emphasis on reciprocal relationships within the community (Aptekar, 2016; Eden, 2017). In contrast, private‐sphere pro‐environmental behaviours are usually driven by personal values, such as biospheric values, concern for the environment or desire to minimize one's carbon footprint. While these values can be shared with others, the actions themselves are typically individual (Tsai et al., 2021). Furthermore, freecycling operates under specific rules and norms, such as not selling items, expressing gratitude and avoiding ‘no‐shows’, which contributes to a moral framework within the community (Aptekar, 2016). In contrast, private‐sphere pro‐environmental behaviours are largely guided by personal beliefs and values, lacking the explicit, community‐enforced norms seen in freecycling (Tsai et al., 2021). Lastly, freecycling represents a form of civic engagement, allowing individuals to view themselves as part of a global digital community that addresses community issues across geographic boundaries (Nelson et al., 2007). In contrast, private‐sphere pro‐environmental behaviours typically do not involve broader civic engagement or collective actions (Tsai et al., 2021). Thus, freecycling stands out not only as the epitome of a non‐monetary and non‐reciprocal sharing economy but also as a unique and powerful form of community‐driven pro‐environmental action that fosters collective responsibility and connection.

### Generalized trust and freecycling

Trust refers to a mindset that embraces vulnerability, grounded in positive expectations about the intentions and behaviours of others (Rousseau et al., 1998). Generalized trust refers to the general willingness to trust others, including strangers, beyond personal relationships (Cao & Galinsky, 2020; Stolle, 2002). Rooted in a moral outlook and optimism about human nature (Uslaner, 2002), generalized trust reflects a cognitive bias towards inferring goodwill in others, even in the absence of concrete information (Yamagishi & Yamagishi, 1994). It fosters the expectation that others will act honestly and benevolently, which serves as a crucial form of social capital that enables cooperation beyond familiar networks. Unlike assurance, which is based on known incentives within committed relationships, generalized trust facilitates credible mutual commitment in socially uncertain contexts, thereby supporting broader civic and economic engagement (Fukuyama, 1995; Putnam, 1993). Generalized trust may enhance pro‐environmental behaviours by potentially alleviating concerns about freeriding. Given that eco‐friendly practices can be costly and effortful (Buder et al., 2014), individuals might be more inclined to participate if they believe others will act similarly. Conversely, a lack of trust can reduce motivation, as people may doubt the effectiveness of their own contributions if others are not doing the same (Gür, 2020). The positive relationship between generalized trust and everyday pro‐environmental behaviours is well‐documented. Individuals with high trust are more likely than those with low trust to choose public transportation (van Lange et al., 1998), purchase eco‐friendly products (Gupta & Ogden, 2009) and recycle (Mannemar Sønderskov, 2011). Higher generalized trust is also associated with reduced consumption of water, energy and disposable items (Gür, 2020).

While generalized trust in private‐sphere pro‐environmental behaviours largely relies on the assumption that some distant or imaginary others are making similar efforts, generalized trust in freecycling is more directly implicated, as the interpersonal dynamics of trust are confronted and negotiated in real time when strangers interact online and meet offline (Aptekar, 2016). In a landscape rife with online scams (INTERPOL, 2024) and considering that the initial interactions in freecycling often occur with anonymous users in online groups, generalized trust is crucial for bridging social distance, making people feel more secure in sharing information with strangers (Nelson & Rademacher, 2009). Additional risks arise when a deal is made, as the actual transaction takes place in real‐world contexts (Norbutas & Corten, 2018). Apart from concerns about personal safety inherent in offline meet‐ups with strangers, a sense of generalized trust is needed for givers to believe their items will be appreciated, used properly and not resold for profit (Eden, 2017; Norbutas & Corten, 2018). Similarly, receivers need to trust that the items offered are safe, hygienic, and in good condition (Özcelik & Kaplan, 2021). Previous qualitative research identified a lack of trust as a major barrier to participating in the sharing economy (Spindeldreher et al., 2019). These small‐sample qualitative studies suggest the need for large‐scale quantitative research to better understand the role of generalized trust in freecycling. Moreover, as trust is largely derived from social experiences (Au et al., 2023), cultural characteristics should also be considered when examining the association of generalized trust with freecycling. Taken together, the following hypothesis was formulated:

Generalized trust will be positively associated with freecycling.

### Climate change conspiracy beliefs and freecycling

Conspiracy theories refer to narratives that explain events – whether historical, current or prospective – by identifying a small group of influential individuals, known as conspirators, who engage in secretive actions for personal benefit or against the interests of society as a whole (Uscinski et al., 2017). Climate change conspiracy beliefs assert that the scientific consensus on climate change is a hoax orchestrated by conspirators, questioning its reality, causation and severity (Chan, Tam, & Hong, 2023; Douglas & Sutton, 2015; Tam & Chan, 2023). These beliefs often target climate scientists, governments and corporations, suggesting motives such as financial profit or political agendas (Douglas & Sutton, 2015; Uscinski & Parent, 2014). While some deny the existence of climate change entirely, others acknowledge it but dispute its anthropogenic causes or downplay its impacts (Smith, 2021; United Nation Environment Programme [UNEP], 2024). Despite the strong scientific consensus on climate change (Cook et al., 2016), a substantial minority – around 17% globally – subscribes to these conspiracy beliefs (YouGov, 2021). This minority stance can result in decreased participation in pro‐environmental behaviours and diminished support for climate policies (Chan, Tam, & Hong, 2023). Such conspiratorial thinking is often linked to a broader tendency to believe in various conspiracy theories and is primarily fuelled by misinformation spread through social media, undermining trust in scientific institutions and hindering effective climate actions (Tam & Chan, 2023). There is a growing consensus in environmental psychology that highlights a critical gap in climate change conspiracy research, which tends to overemphasize Western and developed societies (Biddlestone et al., 2022; Tam & Chan, 2023), underscoring the need for broader geographical representation and cross‐cultural comparisons to fully understand how prevalence these conspiracy beliefs are across diverse cultures.

The negative association of climate change conspiracy beliefs with general pro‐environmental behaviour is well‐documented (Chan, Tam, & Hong, 2023; Wood, 2017). For example, an experimental study found that exposure to climate change conspiracy theories decreases carbon footprint reduction tendencies (Jolley & Douglas, 2014). Moreover, a meta‐analysis of 22 independent studies revealed a moderate‐to‐large negative correlation between climate change conspiracy beliefs and pro‐environmental policy support, and a small‐to‐medium negative correlation with pro‐environmental intentions (Biddlestone et al., 2022).

Although conspiracy beliefs are often viewed as part of a stable, monological mindset, recent research suggests they are fragmented and context‐dependent, shaped by cultural narratives, political climates and situational triggers (Franks et al., 2013; van Prooijen & Douglas, 2017). Rather than subscribing to a broad conspiracist worldview, individuals often endorse specific conspiracy theories selectively, with endorsement patterns influenced by identity, emotional states, perceived threats and media exposure (Imhoff & Lamberty, 2018; Sutton & Douglas, 2020; Wood & Douglas, 2015). This observation suggests the need to reconceptualize conspiracy theories – moving beyond the traditional view of them as a monolithic ideology – and instead viewing conspiracy thinking as a flexible, adaptive response to uncertainty and perceived powerlessness.

Since freecycling represents a unique and emerging community‐based pro‐environmental behaviour, its relationship with climate change conspiracy beliefs remains largely unexplored. This presents two contrasting possibilities that reflect this complex, context‐dependent nature of conspiracy belief outcomes. On one hand, individuals who endorse climate change conspiracy theories may be less inclined to engage in all kinds of pro‐environmental behaviours, including freecycling, due to reduced motivation and increased scepticism towards climate actions, reflecting the well‐established negative association between such beliefs and conventional pro‐environmental behaviours. This scepticism might lead individuals to dismiss freecycling as unnecessary or ineffective. On the other hand, the community‐oriented nature of freecycling may paradoxically appeal to climate change conspiracy believers as a form of grassroots resistance to mainstream narratives, driven by populist or anti‐establishment sentiments (Lewandowsky, 2021). In this way, freecycling could serve as a compelling alternative for those wanting to address hidden environmental issues outside formal institutional channels. By and large, given the relatively stronger empirical evidence linking climate change conspiracy beliefs to reduced general pro‐environmental behaviours, we are inclined to hypothesize a negative association between climate change conspiracy beliefs and freecycling:

Climate change conspiracy beliefs will be negatively associated with freecycling.

Overall, our two hypotheses of H1 and H2 were preregistered before data collection to ensure research transparency and credibility. An exploratory investigation, which was not part of the preregistration, was also conducted to examine the role of societal factors in freecycling participation, as well as their connections to the associations of freecycling with generalized trust and climate change conspiracy beliefs.

### Exploring societal factors in freecycling: Links to generalized trust and climate change conspiracy beliefs

For exploratory purposes, we also examined potential societal factors that may be linked to freecycling and explored how they influence freecycling's associations with generalized trust and climate change conspiracy beliefs. These analyses draw on a socio‐economic‐cultural framework that integrates social and economic development indicators, social beliefs and cultural values – an approach supported by cross‐cultural research on how development, inequality and cultural orientations shape social attitudes and behaviours (Fog, 2023; Gaygısız, 2013; Różycka‐Tran et al., 2015).

Social and economic development often fuels hyperconsumerism, where increased wealth leads to excessive buying and disposability, as well as an unsustainable lifestyle (Toth & Szigeti, 2016). On one hand, aspects of development – such as conscious consumption movements and economic inequality – may support freecycling through selective sustainability and utilitarian demand (Aptekar, 2016; Nelson et al., 2007). On the other hand, in less developed societies, stronger necessity and more equal socio‐economic conditions foster community solidarity and ethical motivations for sharing (Lou, 2019; Norbutas & Corten, 2018; Revinova et al., 2020). While both dynamics may contribute to freecycling, we argue that freecycling is more likely to flourish in contexts with lower levels of social and economic development, where it reflects genuine communal engagement rather than reactive consumption.

Previous research also shows that cultural factors, including social axioms (Leung & Bond, 2004) and cultural values (Hofstede, 2001), can also have differential associations with pro‐environmental behaviours (Chan & Tam, 2021; Mi et al., 2020). Social axioms are generalized beliefs about how the social world functions, comprising five dimensions (Leung & Bond, 2004). Empirical findings show that reward for application (the belief that effort leads to achievement) and social complexity (the belief that human behaviour has multiple causality) are positively associated with pro‐environmental orientation. In contrast, social cynicism (the belief that people and institutions are generally untrustworthy), fate control (the belief that life outcomes are pre‐determined but can still be influenced) and religiosity (the belief in spiritual forces and the importance of religious faith) are negatively associated with pro‐environmental orientation (Chan & Tam, 2021).

Hofstede's five‐dimensional model offers a framework to understand cultural values (Hofstede, 2001). Empirical findings show that long‐term orientation (the focus on future rewards and traditional values) is positively associated with pro‐environmental behaviours in both public‐ and private‐spheres, while individualism (the prioritization of autonomy) and uncertainty avoidance (the intolerance for ambiguity) are negatively associated with them. Masculinity (the emphasis on competition and success) is positively associated with public‐sphere pro‐environmental behaviours, whereas power distance (the acceptance of unequal power distribution) shows no association in either sphere (Mi et al., 2020).

To gain deeper insight into the relationships between broader social, economic and cultural contexts with freecycling, five society‐level covariates were included in our exploratory analyses. These covariates comprise the Human Development Index (HDI; United Nations Development Programme [UNDP], 2025), a comprehensive measure of societal development; the Gini Coefficient (World Bank, 2025), a measure of economic inequality; two sets of Social Axioms Survey (SAS; Leung & Bond, 2004; Stankov & Saucier, 2015); and also Hofstede's Cultural Values (Hofstede, 2001). As advanced and affluent societies tend to adopt unsustainable lifestyle (Toth & Szigeti, 2016), we expected that HDI and Gini Coefficient would be negatively associated with freecycling. Given that freecycling is a pro‐environmental behaviour, we expected that reward for application and social complexity would be positively, whereas social cynicism, fate control and religiosity would be negatively associated with freecycling, based on previous findings (Chan & Tam, 2021). Furthermore, because freecycling involves community engagement that aligns with pro‐environmental behaviours in the public sphere, we anticipated that freecycling would be positively associated with long‐term orientation and masculinity, and negatively with individualism and uncertainty avoidance, as inferred from previous findings (Mi et al., 2020). This multifaceted approach will facilitate a more nuanced understanding of how these societal factors associate with freecycling behaviour.

### The present research

Our research aimed to examine the relationships between generalized trust, climate change conspiracy beliefs and freecycle giving and receiving across 34 countries/societies through the testing of two preregistered hypotheses (preregistration posted on 17 July 2024, prior to data collection; https://osf.io/na4kc/?view_only=543d71e1011749d09d74ad75e6ed81d0).

We collected data from 34 diverse countries/societies across all inhabited continents, with a sample size of 16,773, stratified by age, gender and region of residence to ensure representativeness. Participants came from both developed (e.g. United States and Japan) and developing countries (e.g. Brazil and Indonesia), as well as Western (e.g. Canada and United Kingdom) and non‐Western societies (e.g. China and Nigeria). Our study aimed to address three critical research gaps identified in the literature, including (1) the lack of cross‐cultural comparisons of climate change conspiracy beliefs; (2) the need for large‐scale quantitative research on freecycling; and (3) the importance of incorporating both freecycle giving and receiving. In addition to the preregistered analyses, we also undertook exploratory investigations into freecycling's associations with various society‐level indices and examined how these broader societal factors connect to the relationships of freecycling with generalized trust and climate change conspiracy beliefs, which were not included in the preregistration. Through this comprehensive approach, we aimed to enhance our understanding of the individual and societal factors associated with freecycling, ultimately expanding our knowledge of this unique community‐based pro‐environmental behaviour.

## METHODS

### Participants

We partnered with Kantar, an international data collection company, to gather data from 16,773 adults across 34 countries/societies in Asia, Europe, North America, South America, Oceania and Africa. Using Kantar's extensive participant pool, we employed the panel method to access large, diverse samples, with standardized data collection processes (Hays et al., 2015). We implemented stratified sampling techniques to ensure that the demographic characteristics of the participants, including age, gender and region reflected those in the United Nations (UN) database: http://data.un.org/Host.aspx?Content=About. To maintain data quality, we incorporated three attention check questions (e.g. ‘This is a control question. Select “Agree” and move on’.) throughout the questionnaire (Maniaci & Rogge, 2014). Participants who failed at least one attention check (*n* = 6427) or exhibited systematic response bias (*n* = 263) were excluded. The final valid sample consisted of 16,773 participants (50.7% female, 49.1% male; *M*_age_ = 44.17, SD = 15.07; age range 18–96). Country/society‐level sample sizes ranged from 480 (Turkey) to 509 (Germany). Data were collected between 5 August 2024 and 22 October 2024.

### Procedure

Prior to data collection, we conducted a power analysis to assess the effects of the hypothesized exogenous variables on endogenous variables at the individual level. The analysis indicated that a minimum of 455 participants per country/society was required to achieve 80% statistical power. This estimation was based on a regression model with two independent variables (e.g. generalized trust and climate change conspiracy beliefs) and three covariates (e.g. age, gender and education level). Associations among exogenous variables were assumed to be small‐to‐moderate (ρ = .2), while associations between exogenous and endogenous variables were assumed to be small (ρ = .1; Cohen, 1988). We followed the standard procedures to translate the survey from English into 21 languages and back‐translate each version into English (Brislin, 1986). Any discrepancies noted during the comparison with the original English version were resolved to ensure accuracy. Ethics approval was obtained from the authors' affiliated university, informed consent was sought at the beginning of the survey, and confidentiality was guaranteed to encourage honest response. Demographic information was collected at the end of the survey, and all participants received Kantar points to compensate for their participation.

### Measures

#### Generalized trust

Generalized trust was assessed using an item from the World Value Survey (seventh wave; Haerpfer et al., 2022). Participants were asked to indicate whether they believe most people can be trusted or if one needs to be very careful, rated on a binary scale (1 = *very careful*, 2 = *most people can be trusted*).

#### Climate change conspiracy beliefs

The 3‐item Belief in Climate Change Conspiracy Theories Measure (Chan, Tam, & Hong, 2023) was used to assess beliefs on climate change conspiracy on an 11‐point scale from 0 (*completely false*) to 10 (*completely true*). A sample item is ‘Climate change is a hoax that the government uses to increase taxes paid by citizens’. The Cronbach's alphas for the 34 countries/societies are reported in Table 1.

**TABLE 1:** Cronbach's alpha and measurement invariance testing with alignment optimization procedure.

Group	1	2	3	4	5	6	7	8	9	10	11	12	13	14	15	16	17	18	19	20	21	22	23	24	25	26	27	28	29	30	31	32	33	34	Non‐Invariance
Loading																																			
CCCB1	1	2	3	4	5	(6)	7	8	9	10	11	12	13	14	15	16	17	18	19	20	21	22	23	24	25	26	27	28	29	30	31	32	33	34	1
CCCB2	1	2	3	4	5	6	7	8	9	10	11	(12)	13	14	15	16	17	18	19	20	21	22	23	24	25	26	27	28	29	30	31	32	(33)	34	2
CCCB3	1	2	3	4	5	6	7	8	(9)	10	11	12	13	(14)	15	16	17	18	19	20	21	22	23	24	25	26	27	28	29	30	31	32	33	34	2
PSPEB1	1	2	3	4	5	6	7	8	9	10	11	12	13	14	15	16	17	18	19	20	21	22	23	24	25	26	27	28	(29)	30	31	32	33	(34)	2
PSPEB2	1	2	3	4	(5)	6	7	8	9	(10)	11	(12)	13	14	15	16	17	18	19	(20)	21	22	23	(24)	25	26	27	28	29	30	31	32	33	34	5
PSPEB3	1	(2)	3	(4)	5	6	7	(8)	(9)	10	11	12	(13)	14	15	16	(17)	(18)	19	20	21	22	23	(24)	25	26	(27)	(28)	29	30	31	(32)	(33)	34	12
PSPEB4	1	2	3	4	5	(6)	7	8	9	10	11	12	13	14	(15)	16	17	18	(19)	20	(21)	22	(23)	24	(25)	26	27	28	29	30	(31)	32	(33)	34	8
PSPEB5	1	2	3	4	5	6	7	8	9	10	11	12	13	14	15	16	17	18	19	20	21	22	23	24	25	26	27	28	29	30	31	32	33	34	0
PSPEB6	1	2	3	4	5	6	7	8	9	10	11	12	13	14	15	16	17	18	19	20	21	22	23	24	25	26	27	28	29	30	31	32	33	34	0
PSPEB7	1	2	3	4	5	6	7	8	9	10	11	12	13	14	15	16	17	18	19	20	21	22	23	24	25	26	27	28	29	30	31	32	33	34	0
Intercept																																			32 (9.4%)
CCCB1	(1)	2	3	4	5	6	(7)	8	(9)	10	11	12	13	14	15	(16)	17	18	19	(20)	21	22	23	24	25	26	27	28	29	30	31	32	33	34	5
CCCB2	1	2	3	4	5	6	7	8	9	10	11	12	13	14	15	16	17	18	19	(20)	(21)	22	23	24	25	26	27	28	29	30	31	32	33	34	2
CCCB3	1	2	3	4	(5)	6	7	8	(9)	10	11	12	13	14	15	16	17	18	19	20	21	22	23	24	25	26	27	28	29	30	31	32	(33)	34	3
PSPEB1	1	(2)	3	4	5	6	7	(8)	9	10	11	(12)	(13)	(14)	(15)	(16)	(17)	18	(19)	20	(21)	(22)	23	(24)	25	(26)	27	(28)	(29)	30	31	32	33	(34)	16
PSPEB2	1	2	3	4	5	(6)	7	8	9	10	11	12	13	14	15	16	17	18	19	20	(21)	22	23	24	25	26	27	28	29	30	31	32	33	34	2
PSPEB3	(1)	2	(3)	4	(5)	(6)	7	8	9	10	(11)	(12)	13	14	(15)	(16)	17	18	(19)	(20)	(21)	22	(23)	24	(25)	26	27	28	(29)	30	(31)	32	(33)	(34)	17
PSPEB4	1	2	3	4	5	(6)	(7)	8	9	10	(11)	(12)	13	14	(15)	16	17	(18)	(19)	(20)	(21)	22	(23)	(24)	(25)	(26)	(27)	28	29	(30)	(31)	32	(33)	(34)	18
PSPEB5	1	2	3	4	5	6	7	8	9	10	11	12	13	14	15	16	17	18	19	20	(21)	22	23	24	25	26	27	28	29	30	31	32	33	34	1
PSPEB6	1	2	3	4	5	6	7	8	9	(10)	11	(12)	13	14	15	16	(17)	18	19	20	21	22	23	(24)	(25)	26	27	(28)	29	30	31	32	33	34	6
PSPEB7	1	2	3	4	(5)	(6)	7	(8)	9	10	11	(12)	13	14	15	16	17	18	(19)	(20)	(21)	(22)	23	24	25	26	27	28	(29)	(30)	(31)	32	33	(34)	12
Alpha																																			82 (24.1%)
CCCB	.64	.81	.73	.83	.67	.63	.83	.84	.86	.78	.66	.77	.77	.83	.69	.66	.86	.77	.77	.75	.70	.78	.73	.80	.80	.79	.79	.80	.81	.80	.75	.78	.75	.76	
PSPEB	.78	.75	.79	.70	.76	.79	.71	.72	.70	.78	.73	.79	.71	.72	.77	.78	.75	.78	.71	.80	.82	.71	.80	.79	.76	.73	.74	.71	.68	.80	.81	.72	.77	.69	

*Note*: 1 = Argentina; 2 = Australia; 3 = Brazil; 4 = Canada; 5 = China; 6 = Egypt; 7 = Finland; 8 = France; 9 = Germany; 10 = Hong Kong; 11 = India; 12 = Indonesia; 13 = Italy; 14 = Japan; 15 = Malaysia; 16 = Mexico; 17 = Netherlands; 18 = New Zealand; 19 = Nigeria; 20 = Pakistan; 21 = Philippines; 22 = Portugal; 23 = South Africa; 24 = South Korea; 25 = Singapore; 26 = Spain; 27 = Sweden; 28 = Taiwan; 29 = Thailand; 30 = Turkey; 31 = UAE; 32 = United Kingdom; 33 = USA; 34 = Vietnam.

Abbreviations: CCCB, Climate Change Conspiracy Beliefs; PSPEB, Private‐Sphere Pro‐Environmental Behaviours.

#### Freecycling

A 2‐item Freecycling Scale was used to assess the frequency of engaging in freecycling. Recognizing that the concept of freecycling may lack direct linguistic or cultural equivalents in some societies, a definition was provided before asking participants about their experiences: ‘Freecycling refers to the act of giving items for free to strangers or accepting free items from strangers through social media, online platforms, or mobile apps. The entire transaction process does not involve any exchange of money or items’. Participants were then asked how often they engaged in freecycling – both giving and receiving – in the past 6 months, rated on a 5‐point scale from 1 (*never*) to 5 (*always*).

#### Private‐sphere pro‐environmental behaviours

A 7‐item Private‐Sphere Pro‐environmental Behaviours Scale (Chan, Tam, & Hong, 2023) was used to measure the frequency of eco‐friendly actions taken by respondents in their daily lives over the past week. Responses were rated on a 5‐point scale from 1 (*never*) to 5 (*always*). A sample item is ‘Turn off lights and appliances when not in use’. The Cronbach's alphas for the 34 countries/societies are reported in Table 1.

#### Society‐level indices

Five society‐level indices, namely the Human Development Index 2020–2022 (HDI; UNDP, 2025), Gini Coefficient 2020–2023 (World Bank, 2025), two sets of Social Axioms Survey 2004 (SAS; Leung & Bond, 2004) and 2015 (SAS; Stankov & Saucier, 2015) and Hofstede's Cultural Values (Hofstede, 2001), were included as society‐level variables.

#### Covariates at the individual level

Demographic information (i.e., age, gender and education level), political orientation and social desirability were included as covariates. Political orientation was measured using a single item (Jost, 2006) anchored on a 7‐point scale, ranging from 1 (*very liberal*) to 7 (*very conservative*). The 6‐item Marlowe‐Crowne Social Desirability Scale (MC‐SDS; Fischer & Fick, 1993; Strahan & Gerbasi, 1972) was used to gauge participants' tendency to present themselves in a favourable light. One sample item is ‘I have never intensely disliked anyone’, with dichotomous responses of *true* or *false*.

## RESULTS

### Testing measurement invariance with alignment optimization procedure

Given the multi‐item nature of the measures of climate change conspiracy beliefs and private‐sphere pro‐environmental behaviours, we examined their measurement invariance to enable meaningful comparisons across 34 countries/societies. The traditional multiple‐group confirmatory factor analysis (CFA) approach to establishing configural, metric and scalar invariance often poses difficulties when many groups are involved (Lomazzi, 2018). This approach is typically too strict, making full invariance nearly unattainable as violations of equivalence would increase substantially with the number of groups (Muthén & Asparouhov, 2018). To address this, Asparouhov and Muthén (2014) developed the alignment method as an alternative for handling non‐invariance involving a large number of groups. This method builds on configural invariance testing via multiple‐group CFA and applies an optimization procedure to minimize differences in factor loadings and item intercepts across groups (see Muthén & Asparouhov, 2018). When fewer than 25% of parameters are non‐invariant, factor mean and variance comparisons across groups can be considered trustworthy (Flake & McCoach, 2018; Muthén & Asparouhov, 2018).

As a first step, we fitted a baseline configural model (a correlated factor model of climate change conspiracy beliefs and private‐sphere pro‐environmental behaviour) using multiple‐group CFA. Model fit was assessed using multiple indices following established guidelines (Hoyle, 1995). Acceptable fit was indicated when the Standardized Root Mean Squared Residual (SRMR; Hu & Bentler, 1999) was below 0.08 and the Comparative Fit Index (CFI; Bentler, 1990) exceeded 0.90. Root mean square error of approximation (RMSEA; Browne & Cudeck, 1992; Steiger & Lind, 1980) values were interpreted according to conventional benchmarks: <0.01 reflected excellent fit, 0.01–0.05 close fit, 0.05–0.08 fair fit, 0.08–0.10 mediocre fit and >0.10 poor fit. These criteria have been applied in prior research on measurement invariance and equivalence testing (Cheung & Rensvold, 2002; Yuan et al., 2016). Overall, the configural model showed acceptable fit, χ^2^ (1156) = 5477.89, *p* < .001, CFI = 0.900, SRMR = 0.063, RMSEA = 0.087, supporting configural invariance across the 34 countries/societies. Table 1 presents the second step of alignment optimization results, with numbers in parentheses indicating groups showing non‐invariance for a given parameter. Overall, only 9.4% (32/340) of factor loadings and 24.1% (82/340) of item intercepts were non‐invariant. Using the 25% benchmark, these findings suggest that factor mean and variance comparisons across the 34 countries/societies should be trustworthy and reliable.

### The prevalence of freecycling and its correlations with cultural dimensions across 34 countries/societies

**FIGURE 1:**
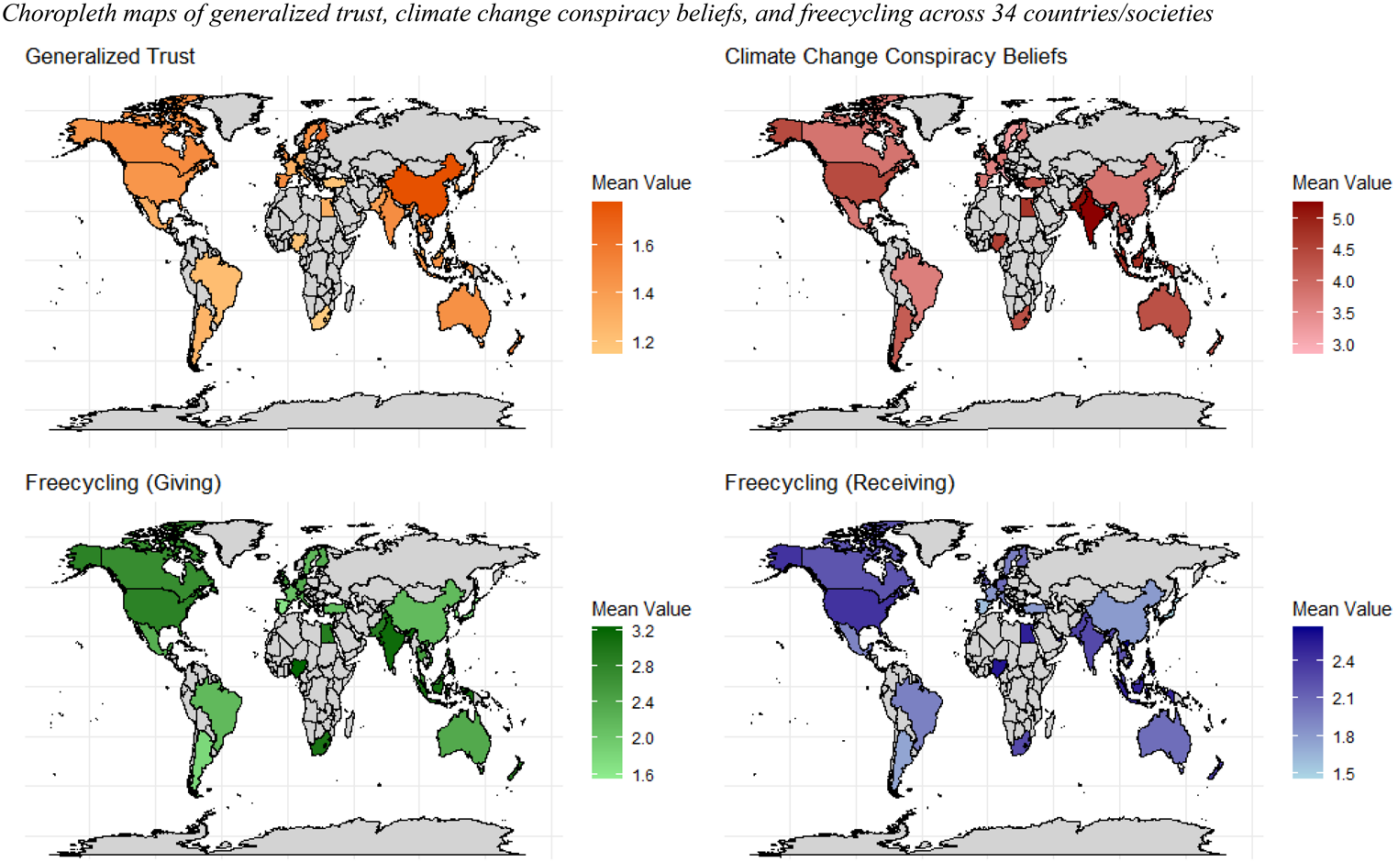
Choropleth maps of generalized trust, climate change conspiracy beliefs and freecycling across 34 countries/societies.

Table 2 presents the societal means of freecycling, complemented by the corresponding choropleth maps shown in Figure 1. Table 3 summarizes the correlations between freecycling and various covariates at the societal level. The Human Development Index (HDI) exhibited a significant negative association with freecycling, indicating that higher HDI is linked to lower levels of freecycling, *r* = −.47, *p* = .005 for giving and *r* = −.41, *p* = .019 for receiving. Furthermore, among the five social axioms, reward for application and religiosity demonstrated consistent positive associations with freecycling at the societal level. These findings are evidenced by two sets of indices from Leung and Bond (2004) and Stankov and Saucier (2015), with *r*s ranging from .43 to .55. Furthermore, within the framework of Hofstede's (2001) cultural values, uncertainty avoidance and long‐term orientation were significantly correlated with lower levels of freecycling, with *r*s ranging from −.42 to −.67.

**TABLE 2:** Societal means of generalized trust, climate change conspiracy beliefs and freecycling across countries/societies.

	*n*	Generalized trust	Climate change conspiracy beliefs	Freecycle giving	Freecycle receiving
		Mean	Mean	Mean	Mean
Argentina	494	1.28	4.17	1.80	1.73
Australia	483	1.47	4.35	2.35	2.05
Brazil	493	1.23	3.66	2.16	1.94
Canada	492	1.50	3.83	2.71	2.20
China	500	1.78	3.81	2.15	1.79
Egypt	498	1.31	4.74	2.91	2.51
Finland	494	1.65	3.63	2.32	2.08
France	494	1.30	3.59	2.05	1.81
Germany	509	1.33	3.78	2.34	2.06
Hong Kong	490	1.51	4.29	2.42	2.07
India	494	1.49	5.26	3.14	2.27
Indonesia	496	1.47	4.85	3.08	2.49
Italy	499	1.32	2.85	2.28	1.91
Japan	492	1.44	3.10	1.54	1.45
Malaysia	493	1.26	4.79	2.61	2.22
Mexico	495	1.32	3.77	2.30	1.92
Netherlands	499	1.55	3.83	2.59	2.12
New Zealand	488	1.56	4.32	2.90	2.36
Nigeria	492	1.20	4.59	3.23	2.57
Pakistan	483	1.38	5.15	2.89	2.30
Philippines	498	1.22	4.77	3.00	2.39
Portugal	492	1.42	4.05	1.87	1.85
South Africa	494	1.15	4.35	3.03	2.26
South Korea	498	1.39	3.47	1.74	1.63
Singapore	492	1.46	4.31	2.69	2.28
Spain	496	1.50	3.79	1.65	1.59
Sweden	495	1.47	3.24	2.16	1.95
Taiwan	493	1.52	4.17	1.95	1.81
Thailand	496	1.45	4.27	2.52	2.19
Turkey	480	1.20	4.29	2.13	1.79
UAE	489	1.30	4.15	3.16	2.60
United Kingdom	493	1.49	3.87	2.62	2.17
USA	483	1.44	4.43	2.84	2.40
Vietnam	496	1.55	3.41	2.63	2.66

**TABLE 3:** Correlations of freecycling with cultural dimensions.

	*n*	Freecycling (giving)	Freecycling (receiving)
		*r*	*r*
Human Development Index	33	−.473**	−.408*
GINI coefficient	22	−.020	−.016
Social cynicism^a^	21	.032	−.034
Social complexity^a^	21	−.334	−.353
Reward for application^a^	21	.465*	.427^†^
Religiosity^a^	21	.489*	.552**
Fate control^a^	21	−.163	−.164
Social cynicism^b^	26	−.300	−.321
Social complexity^b^	26	−.213	−.205
Reward for application^b^	26	.460*	.432*
Religiosity^b^	26	.543**	.563**
Fate control^b^	26	.229	.193
Power distance	31	.078	.059
Individualism (vs. Collectivism)	31	.104	.031
Masculinity (vs. Femininity)	31	.045	−.090
Uncertainty avoidance	31	−.642***	−.668***
Long‐term orientation (vs. Short‐term)	33	−.420*	−.447**
Indulgence (vs. Restraint)	33	.035	.009

*Note*: ^†^ *p* < .10, *p < .05, **p < .01, ***p < .001.

^a^ From Stankov and Saucier (2015).

^b^ From Leung and Bond (2004).

### The associations of generalized trust/climate change conspiracy beliefs with freecycling/pro‐environmental behaviours

The collected data have a multilevel structure, with 16,773 individuals nested within 34 countries/societies. Intraclass correlation coefficients (ICC) were computed for each variable and indicated non‐trivial between‐group variations in this multilevel data. The ICCs range from .04 to .15, with an average of 8% between‐group variation across 34 countries/societies, ${\mathrm{ICC}}_{\mathrm{GT}}$ = .08, ${\mathrm{ICC}}_{\text{CCCB}}$ = .04, ${\mathrm{ICC}}_{\text{PSPB}}$ = .07, ${\mathrm{ICC}}_{\mathrm{FC}-G}$ = .15, ${\mathrm{ICC}}_{\mathrm{FC}-R}$ = .07. To account for this nested data structure, multilevel correlations were first computed to examine the associations among these five constructs (see Table 4). At the individual level, private‐sphere pro‐environmental behaviours were positively correlated with generalized trust, *r* = .10, *p* < .001, and negatively correlated with climate change conspiracy beliefs, *r* = −.06, *p* < .001. These results are consistent with findings from previous research (Gupta & Ogden, 2009; Gür, 2020; Mannemar Sønderskov, 2011; van Lange et al., 1998). Supporting our Hypothesis 1, generalized trust was positively correlated with both freecycle giving, *r* = .09, *p* < .001, and receiving, *r* = .10, *p* < .001. Interestingly, contrary to our Hypothesis 2, we found that climate change conspiracy beliefs were positively correlated with both freecycle giving, *r* = .14, *p* < .001, and receiving, *r* = .18, *p* < .001.

**TABLE 4:** Intercorrelations among constructs at individual and societal levels.

	1	2	3	4	5
1. Generalized trust	–	−.19	−.31	−.16	−.10
2. Climate change conspiracy beliefs	−.06***	–	.46***	.66***	.55***
3. Private‐sphere pro‐environmental behaviours	.10***	−.06***	–	.55***	.41***
4. Freecycle giving	.09***	.14***	.27***	–	.94***
5. Freecycle receiving	.10***	.18***	.17***	.58***	–

*Note*: Intercorrelations at the individual level are displayed below the diagonal, while those at the societal level are shown above the diagonal. Statistical significance (***) for parameter testing was identified when the 95% credible interval (CI) did not cross zero.

Building on the correlation results, a series of multilevel models were estimated using multilevel structural equation modelling (MLSEM), which decomposes variables into within‐ and between‐level components (Muthén, 1994; Muthén & Asparouhov, 2011). The MLSEM was implemented with the Bayesian estimator to generate posterior distributions of parameter values (yielding 95% credible intervals) under non‐informative priors, with a minimum of 5000 and up to 50,000 Markov Chain Monte Carlo iterations across two independent chains. To reduce autocorrelation, only every fifth posterior draw was retained (i.e., thinning = 5). The first half of each chain was discarded as a burn‐in phase, and the second half was retained and combined across chains to form the posterior parameter distributions used for point estimation and statistical inference. Convergence was evaluated using the default potential scale reduction (PSR) criterion of 1.05, where PSR values below 1.05 indicate that estimates from the two chains do not meaningfully differ.

With the above specifications, we tested a multilevel model with random intercepts and fixed slopes across countries/societies (see Table 5). Consistent with the correlation results, private‐sphere pro‐environmental behaviours were positively associated with generalized trust, *β* = .09, 95% CI [0.079, 0.109], and negatively associated with climate change conspiracy beliefs, *β* = −.05, 95% CI [−0.063, −0.034], at the individual level. As in Hypothesis 1, generalized trust was positively associated with both freecycle giving, *β* = .10, 95% CI [0.085, 0.115], and receiving, *β* = .11, 95% CI [0.093, 0.122]. Similar to the correlation results, climate change conspiracy beliefs were positively, rather than negatively, related to both freecycle giving, *β* = 0.15, 95% CI [0.135, 0.164] and receiving, *β* = .18, 95% CI [0.164, 0.193]. This indicated that individuals holding stronger climate change conspiracy beliefs might participate in freecycling to a greater extent. At the societal level, climate change conspiracy beliefs were positively associated with private‐sphere pro‐environmental behaviours, *β* = .37, 95% CI [0.033, 0.652], freecycle giving, *β* = .60, 95% CI [0.318, 0.795] and receiving, *β* = .50, 95% CI [0.169, 0.728].

**TABLE 5:** Multilevel analysis predicting freecycling/private‐sphere pro‐environmental behaviours by generalized trust/climate change conspiracy beliefs.

	Private‐sphere pro‐environmental behaviours	Freecycle giving	Freecycle receiving
	*β* [95% CI]	*β* [95% CI]	*β* [95% CI]
Individual level			
Generalized trust	.**09 [0.079, 0.109]**	.**10 [0.085, .115]**	.**11 [0.093, 0.122]**
Climate change conspiracy beliefs	**−.05 [−0.063, −0.034]**	.**15 [0.135, .164]**	.**18 [0.164, 0.193]**
Societal level			
Generalized trust	−.21 [−0.514, −0.117]	−.03 [−0.329, .252]	.01 [−0.308, 0.310]
Climate change conspiracy beliefs	.**37 [0.033, 0.652]**	.**60 [0.318, .795]**	.**50 [0.169, 0.728]**

Random effect	*b* [95% CI]	*b* [95% CI]	*b* [95% CI]
Generalized trust	.**007 [0.003, 0.015]**	.**021 [0.009, 0.044]**	.**025 [0.012, 0.051]**
Climate change conspiracy beliefs	.**001 [0.001, 0.002]**	.**001 [0.000, 0.001]**	.**001 [0.000, 0.002]**

*Note*: Bold indicates statistical significance based on the 95% credible interval (CI) not containing zero.

The random effects of generalized trust and climate change conspiracy beliefs on outcomes were also examined in the model. All six random slope estimations yielded significant results (see Table 5). Most critically for the central focus on freecycling in this investigation, the associations of generalized trust with freecycle giving, *b* = .021, 95% CI [0.009, 0.044], and freecycle receiving, *b* = .025, 95% CI [0.012, 0.051], showed variations across 34 countries/societies. Likewise, the associations of climate change conspiracy beliefs with freecycling behaviours differed across countries/societies, *b* = .001, 95% CI [0.000, 0.001] for freecycle giving and *b* = .001, 95% CI [0.000, 0.002] for freecycle receiving. Finally, when we included covariates such as age, gender, education level, political orientation and social desirability in the multilevel analysis, the associations of generalized trust and climate change conspiracy beliefs with freecycling behaviours remained largely consistent, except that the association between climate change conspiracy beliefs and private‐sphere pro‐environmental behaviours became non‐significant.

### Unpacking the cultural variations in the associations of generalized trust and climate change conspiracy beliefs with freecycling

Building on the above multilevel model, we specified cross‐level moderation effects to examine whether the five sets of society‐level variables introduced earlier – HDI, the Gini coefficient, two sets of social axioms, and Hofstede's cultural values – could account for cultural variations in the associations of generalized trust and climate change conspiracy beliefs with freecycling. To reduce the risk of overfitting given the limited sample of 34 countries/societies, we first tested the cross‐level moderation effects of each set of society‐level variables separately. We then combined the models by including all significant cross‐level moderators, thereby controlling for their effects simultaneously.

Overall, three society‐level variables – HDI, reward for application, and uncertainty avoidance – showed significant cross‐level moderation effects. First, HDI accounted for cultural variations in the associations of generalized trust with both freecycle giving, *β* = −.737, 95% CI [−1.049, −0.190], and freecycle receiving, *β* = −.446, 95% CI [−0.699, −0.068]. Specifically, in countries/societies with lower HDI values, generalized trust was more strongly associated with freecycling. Second, reward for application demonstrated a similar cross‐level moderation effect, such that in countries/societies with lower levels of reward for application, the association between generalized trust and freecycle giving was stronger, *β* = −.616, 95% CI [−0.956, −0.066]. Finally, uncertainty avoidance significantly explained cultural variations in the associations between climate change conspiracy beliefs and freecycling, *β* = −.444, 95% CI [−0.696, −0.036] for freecycle giving; *β* = −.430, 95% CI [−0.685, −0.040] for freecycle receiving. In other words, in countries/societies with lower levels of uncertainty avoidance, climate change conspiracy beliefs were more strongly associated with both freecycle giving and receiving.

## DISCUSSION

The present study examined the relationships between generalized trust, climate change conspiracy beliefs and freecycling among 16,773 participants across 34 different countries and societies. We tested two preregistered hypotheses to assess these associations. We also conducted exploratory, non–preregistered analyses of how freecycling correlates with various society‐level variables to gain a deeper understanding of the potential connections between broader social, economic and cultural contexts and freecycling. Our research addressed several critical gaps identified in existing literature, making significant contributions to the field. Notably, our study is the first large‐scale cross‐cultural quantitative investigation of freecycling, incorporating both giving and receiving aspects to offer a comprehensive view of this emerging, community‐based pro‐environmental behaviour. Moreover, our study includes cross‐cultural comparisons of climate change conspiracy beliefs, shedding light on how these beliefs may manifest across diverse countries/societies. Our study suggests that generalized trust and climate change conspiracy beliefs are positively associated with freecycling, offering preliminary insights into this unique pro‐environmental behaviour. Moreover, our findings on society‐level associations with freecycling may offer useful implications for policymakers and practitioners engaged in promoting sustainability across diverse cultures. While our findings reveal meaningful associations, the cross‐sectional design limits conclusions about the directionality of these relationships, which should be further explored in future research.

### Associations of generalized trust with freecycle giving and receiving

Consistent with our expectation, generalized trust was positively associated with both freecycle giving and receiving, fully supporting Hypothesis 1. This suggests that individuals who broadly trust the reliability and integrity of others may feel safer and more secure when engaging in exchanges with strangers, thereby facilitating participation in freecycling. These findings align with the perspectives of Fukuyama (1995) and Putnam (1993), who argued that trust supports cooperation and reciprocity in communities. They also resonate with Uslaner (2002) and Yamagishi and Yamagishi (1994), who emphasized that generalized trust facilitates confident interaction with strangers, even in the absence of prior relationships or formal assurances. Moreover, our findings also show that generalized trust is positively associated with private‐sphere pro‐environmental behaviours, consistent with prior research (Gupta & Ogden, 2009; Gür, 2020; Mannemar Sønderskov, 2011; van Lange et al., 1998). Taken together, these findings suggest generalized trust may play a role in supporting engagement in pro‐environmental behaviours, both in everyday life and in community‐based initiatives.

### Associations of climate change conspiracy beliefs with freecycle giving and receiving

Contrary to our expectation, climate change conspiracy beliefs were positively associated, rather than negatively, with both freecycle giving and receiving, failing to support Hypothesis 2. This unexpected result may reflect the nuanced and situational nature of conspiracy beliefs, which – as noted earlier – often emerge in response to perceived institutional distrust and uncertainty (Franks et al., 2013; van Prooijen & Douglas, 2017). One possible explanation is that freecycling might offer a sense of stability and support to individuals dissatisfied with mainstream climate narratives. Previous literature suggests that when people are disappointed by the government and larger systems, they often seek out community connections (Hosking, 2019). Therefore, for individuals who view climate change as exaggerated by powerful others, freecycling could resonate with their grassroots values by offering community‐based alternatives to address environmental challenges. For those who view climate change as a complete hoax, freecycling remains a means for community building, regardless of their environmental stance. It is important to note that our finding also shows climate change conspiracy beliefs were negatively associated with private‐sphere pro‐environmental behaviours, consistent with previous findings (Chan, Tam, & Hong, 2023; Wood, 2017). This contrast underscores the distinctive nature of freecycling as a community‐oriented practice – one that may be perceived as separate from mainstream climate action and thus more acceptable to conspiracy believers. These findings support a growing view that conspiracy thinking reflects a flexible, adaptive response shaped by perceived power structures and institutional distrust (Imhoff & Lamberty, 2018; Sutton & Douglas, 2020).

### Associations of freecycling with society‐level indices

Consistent with our predictions, Human Development Index (HDI) is negatively associated with both freecycle giving and receiving, suggesting that developed societies have lower levels of freecycling compared with developing societies. One explanation for this result is that in more affluent societies, higher disposable incomes decrease reliance on second‐hand goods, fostering a consumer‐oriented culture that prioritizes new purchases over sharing (Campbell, 2014). Cultural attitudes may shift towards individual ownership and consumerism, making freecycling less appealing. Moreover, in these societies, the presence of formal sustainability practices such as structured recycling programs (Mmereki et al., 2016) may reduce the need for grassroots solutions like freecycling.

Also confirming our expectation, the social axiom of reward for application is positively associated with both freecycle giving and receiving. Reward for application suggests believing that effort leads to positive outcomes (Leung & Bond, 2004). Its positive link with freecycling likely stems from the perception that giving contributes to social and environmental good, while receiving can be viewed as a reward for supporting pro‐environmental initiatives. This finding aligns with previous research indicating that reward for application predicts pro‐environmental intention through collective efficacy (Chan & Tam, 2021). However, contrary to our expectation, religiosity is positively, rather than negatively, associated with freecycling. This unexpected finding may be due to the fact that religiosity emphasizes the role of spiritual values in moral behaviour and community building (Leung & Bond, 2004). Its positive association with freecycling may arise from the belief that giving is a moral duty, while receiving is a blessing that reflects community support.

As anticipated, Hofstede's cultural dimension of uncertainty avoidance is negatively associated with both freecycle giving and receiving (Hofstede, 2001). Uncertainty avoidance refers to the extent to which members of a culture feel uncomfortable with ambiguity. Its negative association with freecycling likely stems from a preference for structured exchanges and a sense of distrust towards informal giving and receiving. Specifically, individuals may hesitate to give items due to worries about recipients' intentions and potential risks (Eden, 2017; Norbutas & Corten, 2018), while they may also be reluctant to accept second‐hand goods due to hygiene and quality concerns (Özcelik & Kaplan, 2021). Our finding aligns with previous research showing the negative impact of uncertainty avoidance on pro‐environmental behaviours (Mi et al., 2020).

Unexpectedly, long‐term orientation is negatively, rather than positively, associated with freecycling. Long‐term orientation reflects a culture's emphasis on future rewards, perseverance and thrift. This finding contradicts previous research suggesting that long‐term orientation positively influences pro‐environmental behaviours (Mi et al., 2020), indicating that freecycling is a unique pro‐environmental behaviour that may not align with traditional long‐term motivations. One possible explanation is that in cultures with a strong long‐term orientation, individuals may prioritize planned, strategic investments and savings over spontaneous or community‐based sharing activities like freecycling. These societies might emphasize formal systems and structured approaches to resource management rather than informal, reciprocal exchanges. As a result, freecycling, which often relies on immediate, community‐driven sharing, may be less prevalent. In addition, long‐term oriented cultures may place greater value on personal responsibility and self‐reliance, potentially reducing reliance on communal sharing practices. In contrast, societies with a short‐term orientation might be more open to flexible, immediate solutions such as freecycling, which can provide quick access to needed goods without long‐term commitments. Furthermore, freecycling often thrives in contexts where social bonds and trust within the community are strong and where individuals are motivated by immediate social reciprocity rather than distant future benefits. This dynamic may be more characteristic of cultures with a short‐term orientation, which focus on present relationships and outcomes.

For the connections of broader societal factors to generalized trust and climate change conspiracy beliefs with freecycling, our findings show that the positive associations of generalized trust with both freecycle giving and receiving are stronger in developing than in developed societies. In developing societies, where social networks are often less formalized and economic conditions more precarious, individuals tend to rely heavily on close community or kinship ties (Fukuyama, 1995; Uslaner, 2002). However, for widespread sharing practices like freecycling to flourish, generalized trust – faith in strangers based on shared moral values – is essential. It enables open giving and receiving and fosters cooperation beyond immediate circles, as individuals feel comfortable sharing items, trusting in others' goodwill and believing in a system of generalized reciprocity (Putnam, 1993). This may explain why generalized trust is more strongly associated with freecycling in developing societies, as it helps individuals share beyond close ties, compensating for weaker institutions and fostering sustainable, community‐based exchange.

Our findings also reveal that the positive association of generalized trust with freecycle giving is stronger in societies with lower levels of reward for application. In societies where this belief is strong, individuals may be more inclined to rely on personal effort and merit‐based systems to achieve success, potentially emphasizing individual responsibility over collective or reciprocal actions. In contrast, in societies with lower levels of reward for application, individuals may place greater importance on social trust and communal support to navigate uncertainties and resource limitations (Chen et al., 2016; Ye et al., 2024). In such contexts, generalized trust becomes a crucial social resource that facilitates cooperative behaviours like freecycle giving. When people trust others broadly, they may feel more confident that their generosity will be reciprocated or that the community will support them in times of need, making freecycling a more attractive and viable option. This dynamic suggests that in societies where personal effort is not strongly linked to guaranteed rewards, social trust compensates by fostering collaborative exchanges and mutual aid. Freecycle giving, which relies on voluntary sharing without direct monetary compensation, may thus flourish in environments where trust in others is essential for social and material support.

Our findings indicate that the positive links between climate change conspiracy beliefs and both freecycle giving and receiving are significantly stronger in societies with lower uncertainty avoidance. In these cultures, individuals are more comfortable with ambiguity, fostering openness to informal exchanges and sharing. This comfort enables individuals to more easily navigate concerns about recipients' intentions and potential risks (Eden, 2017; Norbutas & Corten, 2018), as well as worries about hygiene related to second‐hand goods (Özcelik & Kaplan, 2021). This willingness may enhance freecycling participation as a proactive response to climate change conspiracy beliefs, linking their beliefs to community‐oriented actions that challenge mainstream narratives.

### Implications

Our research has two key methodological implications for freecycling and climate change conspiracy research. First, our study advances the understanding of freecycling through a comprehensive methodological framework. It represents the first large‐scale cross‐cultural quantitative investigation of freecycling, explicitly incorporating both giving and receiving aspects to provide a comprehensive understanding of this emerging community‐based pro‐environmental behaviour. We assembled a robust and representative sample from 34 diverse countries/societies across all inhabited continents, encompassing both developed and developing, as well as Western and non‐Western contexts. This approach helps address the important gap in existing research – largely small‐scale, single‐culture, qualitative and focused only on giver experiences. By integrating diverse cultural perspectives, our study sets a foundation for future research, supporting a deeper understanding of freecycling dynamics globally and providing a robust framework for comparing findings across different contexts and cultures.

Second, our study adds to the understanding of climate change conspiracy beliefs through cross‐cultural comparisons. It includes an examination of how these beliefs manifest across diverse countries and societies, responding to the call for broader geographical representation and cross‐cultural comparisons of these conspiracy beliefs across cultures. Moreover, our findings suggest a challenge to existing views on climate change conspiracy beliefs by indicating that, although these beliefs are often associated with decreased pro‐environmental behaviours in private spheres, they may also be linked to unexpected positive engagement in freecycling. This points to possible directions for future research to explore this intriguing aspect of conspiracy beliefs.

Our research also has two important practical implications for enhancing pro‐environmental engagement. First, our study highlights the potential of fostering local community engagement to connect climate change conspiracy believers. Individuals who believe in climate change conspiracies may feel disconnected from broader pro‐environmental movements, making global initiatives less effective. To encourage freecycling among this demographic, the primary focus should be placed on the local community. This can include arranging visits to local landfills to highlight waste issues and organizing clean‐up activities to foster collective responsibility. Emphasizing the immediate benefits of freecycling such as saving money and reducing waste may also resonate more deeply and motivate engagement among climate change conspiracy believers.

Second, our study offers insights into targeted initiatives to foster freecycling across diverse cultural contexts. To promote freecycling across various cultural contexts, initiatives should focus on enhancing community values and addressing specific cultural attitudes. Campaigns can leverage the positive associations of social axioms like reward for application and religiosity, emphasizing that giving and receiving contribute to social good and community support. Furthermore, tailored strategies that alleviate concerns related to uncertainty avoidance, such as establishing structured platforms for safe exchanges, could help build trust in informal giving and receiving. By framing freecycling as a beneficial practice that offers immediate satisfaction and community engagement, these initiatives may be more likely to resonate with diverse cultural norms and encourage participation.

### Limitations and future directions

Despite the merits of our study in methodological and practical contributions, there are several limitations to acknowledge that can guide future studies. A key limitation of our study is its cross‐sectional design, which captures data at a single time point and restricts our ability to draw robust conclusions about directionality or causality. While our models examined associations between generalized trust, climate change conspiracy beliefs and freecycling across 34 cultures, we interpret these findings as correlational rather than causal. To advance this line of inquiry, future research should adopt cross‐lagged longitudinal designs, which allow for the examination of reciprocal relationships over time and improve causal identification by leveraging temporal precedence (Chan, Hong, & Lin, 2023; Rohrer et al., 2022).

In addition, future studies should explore the psychological and social mechanisms that may underlie the observed links between generalized trust and freecycling, and between climate change conspiracy beliefs and freecycling. Several constructs warrant consideration as potential mediators. One possibility is alienation. Individuals who distrust institutional actors – such as governments or corporations – may feel disconnected from official climate narratives and instead turn to grassroots or community‐based sustainability efforts that align with alternative worldviews, potentially increasing their engagement in freecycling (Ifeagwazi et al., 2015; van Prooijen & Douglas, 2017). Another candidate is group identity. People who are generally trusting may be more likely to perceive others as cooperative and morally aligned, which can foster identification with environmental communities and promote shared norms that support sustainable action in the public sphere (Marchlewska et al., 2018; Xing et al., 2022). Uncertainty reduction may also play a role. Trusting individuals may experience less ambiguity and risk in peer‐to‐peer exchanges, while those who endorse conspiracy beliefs may be motivated to take personal responsibility through direct action due to scepticism about institutional solutions (Chan, Tam, & Hong, 2023; Yamagishi & Yamagishi, 1994). Finally, psychological distress may shape behavioural responses to cues to climate change conspiracies, potentially intensifying emotional readiness and urgency to engage in freecycling (Biddlestone et al., 2022; Chan, Tam, & Hong, 2023; Pekárová, 2021).

Our analyses show that the cross‐level moderation effects are medium in size, suggesting that HDI, reward for application, and uncertainty avoidance meaningfully contribute to, but do not fully explain, the strength of the associations of generalized trust and climate change conspiracy beliefs with freecycling. While these moderators capture important cultural variation, future studies should examine other moderators that may account for more substantial variance in random slopes across countries/societies. Given the cross‐cultural scope of our dataset, future research could also benefit from testing cross‐level moderated mediation models to examine whether cultural‐level factors moderate individual‐level mediation pathways, which require longitudinal data to establish directionality and reduce the risk of spurious associations (Chan, Tam, & Hong, 2023; Rohrer et al., 2022). Such designs can offer a more nuanced understanding of how cultural context shapes the psychological processes underlying freecycling behaviour and help identify culturally sensitive strategies for promoting environmental participation.

Moreover, while the use of two frequency items in the current study provides a quantitative snapshot of participation, freecycling is a multifaceted activity embedded in online platforms (Böcker & Meelen, 2017), influenced by item value perceptions and shaped by community norms (Aptekar, 2016; Böcker & Meelen, 2017). These contextual factors contribute to a more nuanced understanding of the behaviour, and frequency measures, while useful, may not capture its full complexity. Future research could benefit from incorporating in‐depth approaches such as textual analysis of posts and participant observations, as well as developing multi‐item scales to assess how users navigate the online environment, adhere to or influence community norms or perceive item value beyond monetary worth. Similarly, our use of a single binary item to assess generalized trust may not fully reflect the multidimensional nature of trust, such as particularistic trust, non‐familial particularized trust or outgroup trust. Future research should employ multi‐item, multidimensional measures of trust – such as those available in the World Values Survey (Wave 7) – to disentangle how different dimensions of trust may relate to freecycling.

Lastly, despite the strengths of our large‐scale cross‐cultural study, its quantitative nature is inherently inadequate to capture the rich, contextual insights crucial for understanding participants' experiences related to freecycling, risking the oversight of essential cultural nuances and individual perspectives. To enhance the robustness of future research, we recommend using a mixed‐methods approach that incorporates qualitative interviews in each society to deepen our understanding of the dynamics among generalized trust, climate change conspiracy beliefs, and freecycling, thereby enhancing the validity of findings and effectively address the complexities of these relationships.

## CONCLUSIONS

Our study highlights the intricate relationships between generalized trust, climate change conspiracy beliefs and freecycling behaviours across 34 cultures. Our findings underscore that generalized trust was positively associated with freecycling, while climate change conspiracy beliefs also showed a positive association with engagement in this community‐driven initiative. By addressing the gaps in existing literature, particularly the need for cross‐cultural comparisons, our research contributes valuable insights into the variables associated with freecycling participation. This understanding can inform targeted initiatives to foster pro‐environmental engagement and community building. As we navigate the challenges of hyperconsumerism and climate change conspiracy beliefs, promoting freecycling may represent a promising strategy to support sustainability and strengthen community ties, potentially contributing to a more resilient and environmentally conscious society.

## AUTHOR CONTRIBUTIONS

**Algae K. Y. Au:** Project administration; conceptualization; methodology; writing – original draft; writing – review and editing. **Jacky C. K. Ng:** Conceptualization; formal analysis; methodology; writing – original draft; writing – review and editing. **Sylvia Xiaohua Chen:** Conceptualization; funding acquisition; supervision; methodology; writing – review and editing.

## CONFLICT OF INTEREST STATEMENT

The authors declare no competing interests.

## ETHICS STATEMENT

Approval was granted by the Human Subjects Ethics Sub‐Committee of the Hong Kong Polytechnic University (No. HSEARS20240728002). This study was performed in accordance with relevant guidelines/regulations. Research involving human research participants was performed in accordance with the Declaration of Helsinki.

## Data Availability

The data and codes that support the findings of this study are available from the authors upon reasonable request.
